# Supplementary material for: Skull morphology diverges between urban and rural populations of red foxes mirroring patterns of domestication and macroevolution
Source: Proc Biol Sci. 2020 Jun 3;287(1928):20200763. doi: 10.1098/rspb.2020.0763 (PMC7341913; doi:10.1098/rspb.2020.0763)
Supplement: Supplemental Tables S1 - S4 [file RSPB20200763supp1.pdf]

Supplemental Table 1. Specimen identification numbers along with sex, habitat classification, and location information for each V. Vulpes used. Specimens were obtained from the National Museums Collections Centre in Edinburgh, Scotland.

Specimen ID#	sex	habitat	latitude	longitude
	f	Rural	51.457089	0.049663782
1284	f	rural	51.523	-0.709
1300	f	rural	51.384735	-0.010845
1299	f	rural	51.4243	0.05892
1289	f	rural	51.4243	0.05892
1318	f	rural	51.424438	0.157519
1316	f	rural	51.424438	0.157519
1315	f	rural	51.422438	0.157519
1302	f	rural	51.338299	-0.0463
1339	f	rural	51.398	-0.63
1332	f	rural	51.3	0.2
1329	f	rural1259	51.45096	-0.293072
1328	f	rural	51.434829	-0.308003
1345	f	rural	51.3	0.2
1376	f	rural	51.433625	-0279207
1630	f	rural	51.403534	0.18667001
1632	f	rural	51.362269	0.064909458
1650	f	rural	51.363327	0.06678700
1653	f	rural	51.431859	0.078320503
1258	m	rural	51.457089	0.049663782
1257	m	rural	51.633	0.355
1256	m	rural	51.278	0.1846
1321	m	rural	51.424438	0.157519
1320	m	rural	51.424438	0.157519
1319	m	rural	51.424438	0.157519
1317	m	rural	51.424438	0.157519
1309	m	rural	51.394332	-0.334396
1618	m	rural	51.518878	0.41218042
1631	m	rural	51.362269	0.064909458
1634	m	rural	51.362269	0.064909458
1633	m	rural	51.362269	0.064909458
1645	m	rural	51.412845	0.35679817
1646	m	rural	51.412845	0.35679817
1642	m	rural	51.363327	0.066787004
1641	m	rural	51.363327	0.066787004
1263	f	urban	51.58659	-0.402997
1262	f	urban	51.604909	0.41003772
1261	f	urban	51.384376	0.51592300
1260	f	urban	51.372361	0.0666
1283	f	urban	51.402044	0.040641
1281	f	urban	51.376341	-0.024912
1278	f	urban	51.368686	0.112417
1274	f	urban	51.600667	-0.2602
1266	f	urban	51.434053	0.175588
1297	f	urban	51.376077	0.003616
1295	f	urban	51.432534	0.04894495
1292	f	urban	51.432534	0.048944950

1287	f	urban	51.42434	0.058922
1286	f	urban	51.422494	0.060381889
1311	f	urban	51.659129	-0.203281
1307	f	urban	51.659129	-0203281
1335	f	urban	51.581283	-0.441964
1324	f	urban	51.348386	-0.165439
1351	f	urban	51.659129	-0.203281
1350	f	urban	51.599698	-0.409123
1371	f	urban	51.35916	-0.198569
1369	f	urban	51.352313	0.089886
1571	f	urban	51.572183	-0.426082
1553	f	urban	51.457467	0.077977180
1562	f	urban	51.452576	0.29362649
1567	f	urban	51.446420	0.054495050
1565	f	urban	51.400541	-0.017412901
1566	f	urban	51.427203	-0.062699318
1570	f	urban	51.564888	-0.34306899
1568	f	urban	51.446420	-0.05449505
1583	f	urban	51.587105	-0.37798500
1578	f	urban	51.435006	0.02636611
1599	f	urban	51.361585	0.066183507
1613	f	urban	51.659129	-0.20328100
1612	f	urban	51.378039	0.018292665
1614	f	urban	51.659129	-0.20328100
1635	f	urban	51.584776	-0.37415585
1644	f	urban	51.659129	0.20328100
1253	m	urban	51.398965	-0.048859119
1251	m	urban	51.365451	-0.15223950
1247	m	urban	51.378039	0.01829266
1282	m	urban	51.376341	-0.024912
1280	m	urban	51.578776	-0.398329
1267	m	urban	51.342315	-0.583498
1296	m	urban	51.376077	0.003616
1293	m	urban	51.432534	0.048945
1290	m	urban	51.432534	0.048945
1312	m	urban	51.659129	-0.203281
1310	m	urban	51.421664	-0.285956
1337	m	urban	51.581283	-0.441964
1327	m	urban	51.659129	-0.203281
1326	m	urban	51.63175	0.04201
1352	m	urban	51.578453	-0.397159
1348	m	urban	51.659129	-0.203281
1347	m	urban	51.475610	0.082783699
1377	m	urban	51.594975	-0.41936874
1546	m	urban	51.610230	-041423923
1478	m	urban	51.602759	-0.43381512
1580	m	urban	51.545253	-0.438102601589
1589	m	urban	51.659129	-0.20328100
1582	m	urban	51.396663	0.04869550
1585	m	urban	51.390227	-0.003735
1586	m	urban	51.594975	-0.41936874

1590	m	urban	51.451413	0.015200542
1594	m	urban	51.659129	-0.2032810
1598	m	urban	51.361585	0.066183507
1597	m	urban	51.353568	-0.17653180
1581	m	urban	51.457467	0.07797718
1601	m	urban	51.597674	-0.3916050
1610	m	urban	51.597674	-0.3916050
1611	m	urban	51.399969	0.01026548
1624	m	urban	51.659129	-0.2032810
1625	m	urban	51.388384	-0.22764444
1637	m	urban	51.396629	0.0490240
1640	m	urban	51.659129	-0.2032810
1648	m	urban	51.573976	-0.46932220

Supplemental Table 2. Anatomical definitions of landmark points used to measure the shape of fox skulls.

Dorsal aspect

1. Anterior tip of the bony septum between the upper central incisors and midline of the premaxilla
2. Premaxillary/maxillary suture, anterior right side
3. Premaxillary/maxilla suture, anterior tip, right side
4. Nasal, anterior tip, right side
5. Nasal, midline
6. Nasal, anterior tip, left side
7. Premaxillary/maxillary suture, anterior left side
8. Premaxillary/maxillary suture, anterior tip, left side
9. Maxilla indentation, right side
10. Premaxillary/maxillary suture, posterior end, right side
11. Premaxillary/maxillary suture, posterior end, left side
12. Maxilla indentation, left side
13. Frontal/maxillary/nasal suture, right side
14. Frontal/maxillary/nasal suture, left side
15. Zygo/maxillare inferior, right side
16. Frontal/maxillary suture, posterior, right side
17. Nasion, nasal/frontal suture, midline
18. Frontal/maxillary suture, posterior, left side
19. Zygo/maxillare inferior, left side
20. Squamosal/jugal suture, anterior projection of the zygomatic process of the temporal bone, right side
21. Occipital tip of frontal bone, right side
22. Occipital tip of frontal bone, left side
23. Squamosal/jugal suture, anterior projection of the zygomatic process of the temporal bone, left side
24. Squamosal/jugal suture, posterior projection of jugal, right side
25. Frontal/parietal/sphenoid suture, right side
26. Bregma, frontal/parietal suture, midline
27. Frontal/parietal/sphenoid suture, left side
28. Squamosal/jugal suture, posterior projection of jugal, left side
29. Squamosal- posterior junction of the zygomatic arch and parietal, right side
30. Squamosal- posterior junction of the zygomatic arch and parietal, left side
31. External auditory meatus, posterior, right side
32. Lambda, parietal/occipital suture, midline
33. External auditory meatus, posterior, left side
34. Asterion, posterior at occipital/parietal/temporal suture, right side
35. Occipital bone, posterior midline
36. Asterion, posterior at occipital/parietal/temporal suture, left side

Ventral aspect

1. Inferior tip of the bony septum between the upper central incisors, midline
2. Canine (anterior buccal corner), left side
3. Canine (anterior buccal corner), right side

4. Canine (posterior buccal corner), left side
5. Premaxillary/maxillary suture, posterior, midline
6. Canine (posterior buccal corner), right side
7. Premolar 4 (posterior buccal corner), left side
8. Palatine/maxillary suture, right side
9. Maxillary/palatine suture, anterior, midline
10. Palatine/maxillary suture, left side
11. Premolar 4 (posterior buccal corner), right side
12. Palatine, posterior, midline
13. Squamosal/jugal suture, anterior projection of the zygomatic process of temporal bone, left side
14. Presphenoid, anterior tip, midline
15. Squamosal/jugal suture, anterior projection of the zygomatic process of temporal bone, right
16. Anterior indent, frontal, left side
17. Presphenoid/basisphenoid suture, midline
18. Anterior indent, frontal, right side
19. Squamosal/jugal suture, posterior projection of jugal, right side
20. Frontal/parietal/sphenoid suture, left side
21. Frontal/parietal/sphenoid suture, right side
22. Squamosal/jugal suture, posterior projection of jugal, left side
23. Auditory bulla, posterior tip, left
24. Tympanoccipital fissure, anterior tip, left side
25. Tympanoccipital fissure, anterior tip, right side
26. Auditory bulla, posterior tip, right
27. Occipital condyle (widest point of foramen magnum), left side
28. Opisthion, dorsal lip of foramen magnum, midline
29. Occipital condyle (widest point of foramen magnum), right side

Supplemental Table 3. Sample sizes for each of the Vulpini species and close relatives used for morphological landmarks.

Species	Sample size
Otocyon megalotis	27
Nyctereutes procyonoides	10
Vulpes bengalensis	26
Vulpes cana	4
Vulpes chama	15
Vulpes corsac	4
Vulpes ferrilata	4
Vulpes macrotis	1
Vulpes lagopus	31
Vulpes rueppellii	27
Vulpes velox	2
Vulpes zerda	11

Supplemental table 4. Variation explained by the first six principal components derived from the dorsal and ventral aspects of the genus *Vulpes*.

Individual dorsal PC Summary

	PC1	PC2	PC3	PC4	PC5	PC6
Standard deviation	0.02207	0.01761	0.01678	0.01419	0.0112	0.0108
Proportion of Variance	0.21519	0.13694	0.12438	0.08899	0.0554	0.05149
Cumulative Proportion	0.21519	0.35213	0.4765	0.56549	0.6209	0.67238

Mean dorsal PC Summary

	PC1	PC2	PC3	PC4	PC5	PC6
Standard deviation	0.02123	0.01809	0.01426	0.01341	0.01177	0.009814
Proportion of Variance	0.29982	0.21786	0.13526	0.11964	0.09223	0.06409
Cumulative Proportion	0.29982	0.51768	0.65293	0.77257	0.8648	0.92889

Individual ventral PC Summary

	PC1	PC2	PC3	PC4	PC5	PC6
Standard deviation	0.03156	0.01832	0.01311	0.0119	0.01049	0.00978
Proportion of Variance	0.38524	0.12975	0.06644	0.05472	0.04253	0.03699
Cumulative Proportion	0.38524	0.51499	0.58143	0.63616	0.67869	0.71568

Mean ventral PC Summary

	PC1	PC2	PC3	PC4	PC5	PC6
Standard deviation	0.03109	0.01735	0.01338	0.01218	0.009	0.00805
Proportion of Variance	0.5268	0.16405	0.09763	0.08093	0.04416	0.03533
Cumulative Proportion	0.5268	0.69086	0.78848	0.86942	0.91357	0.9489
